# Inferring the risk factors behind the geographical spread and transmission of Zika in the Americas

**DOI:** 10.1371/journal.pntd.0006194

**Published:** 2018-01-18

**Authors:** Lauren M. Gardner, András Bóta, Karthik Gangavarapu, Moritz U. G. Kraemer, Nathan D. Grubaugh

**Affiliations:** 1 School of Civil and Environmental Engineering, UNSW Sydney, Sydney, New South Wales, Australia; 2 Department of Immunology and Microbial Sciences, The Scripps Research Institute, La Jolla, California, United States of America; 3 Department of Zoology, University of Oxford, Oxford, United Kingdom; 4 Boston Children’s Hospital, Boston, Massachusetts, United States of America; 5 Harvard Medical School, Boston, Massachusetts, United States of America; DoD - AFHSB, UNITED STATES

## Abstract

**Background:**

An unprecedented Zika virus epidemic occurred in the Americas during 2015-2016. The size of the epidemic in conjunction with newly recognized health risks associated with the virus attracted significant attention across the research community. Our study complements several recent studies which have mapped epidemiological elements of Zika, by introducing a newly proposed methodology to simultaneously estimate the contribution of various risk factors for geographic spread resulting in local transmission and to compute the risk of spread (or re-introductions) between each pair of regions. The focus of our analysis is on the Americas, where the set of regions includes all countries, overseas territories, and the states of the US.

**Methodology/Principal findings:**

We present a novel application of the Generalized Inverse Infection Model (GIIM). The GIIM model uses real observations from the outbreak and seeks to estimate the risk factors driving transmission. The observations are derived from the dates of reported local transmission of Zika virus in each region, the network structure is defined by the passenger air travel movements between all pairs of regions, and the risk factors considered include regional socioeconomic factors, vector habitat suitability, travel volumes, and epidemiological data. The GIIM relies on a multi-agent based optimization method to estimate the parameters, and utilizes a data driven stochastic-dynamic epidemic model for evaluation. As expected, we found that mosquito abundance, incidence rate at the origin region, and human population density are risk factors for Zika virus transmission and spread. Surprisingly, air passenger volume was less impactful, and the most significant factor was (a negative relationship with) the regional gross domestic product (GDP) per capita.

**Conclusions/Significance:**

Our model generates country level exportation and importation risk profiles over the course of the epidemic and provides quantitative estimates for the likelihood of introduced Zika virus resulting in local transmission, between all origin-destination travel pairs in the Americas. Our findings indicate that local vector control, rather than travel restrictions, will be more effective at reducing the risks of Zika virus transmission and establishment. Moreover, the inverse relationship between Zika virus transmission and GDP suggests that Zika cases are more likely to occur in regions where people cannot afford to protect themselves from mosquitoes. The modeling framework is not specific for Zika virus, and could easily be employed for other vector-borne pathogens with sufficient epidemiological and entomological data.

## Author summary

Since May 2015, when Zika was first reported in Brazil, the virus has spread to over 60 countries and territories, and imported cases of Zika have been increasingly reported worldwide. However, there is still much uncertainty behind the mechanisms which dictated the rapid emergence of the epidemic. This work introduces a novel modeling framework to improve our understanding of the risk factors which contributed to the geographic spread and local transmission of Zika during the 2015-2016 epidemic in the Americas. The model is informed by data on regional socioeconomic factors, mosquito abundance, travel volumes, and epidemiological data. As expected, our results indicate that increased presence of mosquitoes, human hosts, and viruses increase the risk for mosquito-borne virus transmission. Passenger air travel, however, was less impactful, suggesting that travel restrictions will have minimal impact on controlling similar epidemics. Importantly, we found that a lower regional GDP was the best predictor of Zika virus transmission, suggesting that Zika is primarily a disease of poverty.

## Introduction

Prior to 2015, local cases of Zika virus had only been reported in Africa and Asia, most prominently in the Pacific Islands [1]. Phylogenetic analysis suggest the virus was introduced into the Americas as early as 2013 [2, 3], but it was not detected in Brazil until May 2015. By this time, Zika virus had already silently spread throughout most of the Americas [3, 4]. As of 2016, local Zika virus transmission has been established in over 60 countries and territories, with the number of estimated cases exceeding 750 thousand [5]. Zika virus infection typically presents with mild flu like symptoms, and in many cases the infection is asymptomatic. However, the potential harm posed by Zika is now known to be substantially greater since it has been associated with a rare congenital disease, microcephaly [6–13], and Guillain-Barre syndrome [13]. The unprecedented size of the outbreak and links to severe disease prompted the WHO to declare the current Zika virus outbreak a public health emergency of international concern [14]. The emergency status lasted until November 2016, at which point Zika virus was recognized to remain a significant enduring public health challenge [15].

Like dengue and chikungunya, Zika is a vector-borne virus primarily transmitted by *Aedes aegypti* [16–22]. Geographic spread of the viruses occurs through global transport systems, such as passenger air travel, cruises, and maritime freight, where infected travelers depart affected regions for destinations where competent vector species have established populations [2, 23–26]. The numbers of recent travel-related Zika cases diagnosed around the world (*e.g.,* United States, Europe, Australia, New Zealand and China) [27] demonstrates how these networks facilitate virus emergence in new areas. It is not always clear, however, what factors are necessary for successful establishment and outbreaks. For example, in many countries, imported cases did not result in local transmission, while phylogenetic analysis shows that some regional outbreaks were initiated by multiple Zika virus introductions [3, 4, 24, 28].

The objective of our work is to better understand the risk factors which contributed to the spread of Zika virus during the 2015-2016 epidemic in the Americas. Our work complements and builds upon several recent studies investigating the potential spread of Zika into new regions by utilizing a substantially different framework. Monaghan *et. al.* [29] overlaid simulated *Ae. aegypti* and *Ae. albopictus* mosquito abundances, travel capacities, and socioeconomic factors to estimate the cities in the United States with the highest expected cases of travel-imported Zika. In two studies, Bogoch *et. al.* [30, 31] presented the potential for Zika virus spread into the rest of the Americas, Africa, and the Asia Pacific region using air travel and vector abundance risk maps. Nah *et. al.* [32] used survival analysis and publicly available epidemiological and air travel data to predict the risk of importation and local transmission of Zika virus at the country level. Zhang *et. al.* [33] applied a stochastic epidemic model to simulate the spatiotemporal spread of the virus at a global scale, and estimated both the number of Zika infections and microcephaly for several countries in the Americas. Ogden *et. al.* [34] demonstrated that risk to travelers in Zika virus affected countries correlates with estimates of R0 using human case surveillance data. Others have also estimated R0 to assess transmission risk across a variety of environments [35–37]. We previously mapped the variations in the geographic risk profile under different assumptions of vector species capacities for Zika virus transmission [38], and illustrated the geographic spread of Zika virus to be driven primarily by *Ae. aegypti* [39]. In collaboration with a large international research team, we also analyzed sequenced Zika virus genomes, reported cases, mosquito abundance, and travel patterns to track the spread of the epidemic (*i.e.,* genomic epidemiology) [3, 4, 24].

The proposed methodology we present in this paper is what we refer to as the Generalized Inverse Infection Model (GIIM), initially introduced in [40], and further expanded upon in [41]. GIIM is a network optimization model originally designed for static environments, which we extended to a dynamic framework. Here, we define the network structure by the passenger air travel movements between all pairs of regions in the Americas. For each region, we provide the model data regarding socioeconomic factors, available health infrastructure, vector habitat suitability, passenger air travel, and reported Zika case numbers. Our model estimates the relative contribution of each risk factor in the spread and local transmission of Zika virus using a multi-agent based optimization search method to estimate the parameters of a link risk function. Specifically, our results provide quantitative time-dependent estimates for the likelihood of Zika virus spreading between regions, resulting in local transmission at the destination. At a regional level, exportation, and importation risk profiles are also provided. The risk function parameters are estimated through iterative refinement, the direction of which is driven by an error function between predicted and observed properties of an infection process, similar to what we previously implemented for dengue virus [25]. Our results indicate that the most common transmission routes were to countries with some of the lowest gross domestic product (GDP) per capita in the Western Hemisphere [42].

### Data

The proposed model is evaluated at a country level, and therefore dependent on country level data for input. The socioeconomic data, epidemiological data, travel data, and vector suitability data for the principle spreading vectors species are all aggregated to the country level for all countries and territories in the Americas as well as the individual U.S. States. Each variable considered in the model is described in further detail below. All data that we can make publicly available are listed in S1 Data.

#### Epidemiological data

**Fig 1:**
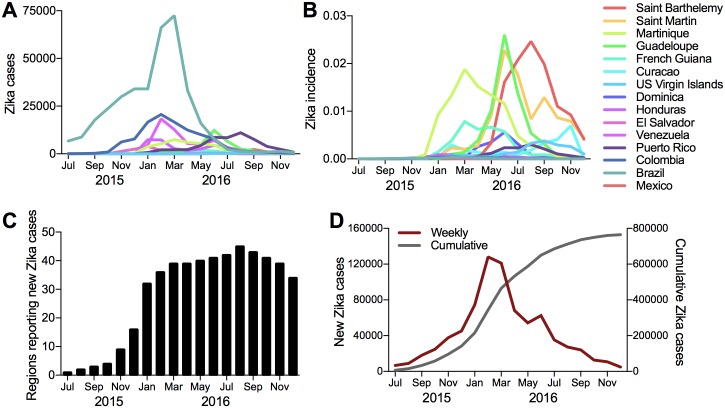
Reported suspected and confirmed Zika cases in the Americas. Monthly distribution of reported (A) Zika cases and (B) Zika incidence rates for 15 countries and territories during the 2015-2016 epidemic (listed in descending order of incidence rates). (C) The number of countries and territories reporting local Zika cases per month. (D) The total number of Zika cases reported in the Americas per month and cumulatively during the epidemic.

We collected the weekly suspected and confirmed Zika cases from each each country and territory in the Americas from the Pan American Health Organization (PAHO) [43], as we previously described [24] (data available: github.com/andersen-lab/Zika-cases-PAHO). Here, we aggregated case to the monthly level for use in our model (Fig 1). The monthly country level case counts were divided by each country population to compute incidence rates for use in the analysis. We chose to use incidence rates (Fig 1B) rather than case counts in the model, as incidence rates more accurately capture the likelihood of an individual traveler being infected.

Although the first Zika cases were reported in Brazil in May 2015, no case data is available for all of 2015 from PAHO because Zika cases and associated neurological and congenital syndrome were not made notifiable conditions by the Brazil Ministry of Health until 17 February of 2016 [43]. Given the lack of case data yet confirmed circulation of Zika in Brazil, we estimated the number of reported cases from July to December 2015 to increase proportionally to the monthly suitability during that time period, and calculated the values as a function of the reported January 2016 case counts. This approach was selected based on the positive correlation between *Ae. aegypti* abundance and reported case counts in Brazil [2].

#### Travel data

**Fig 2:**
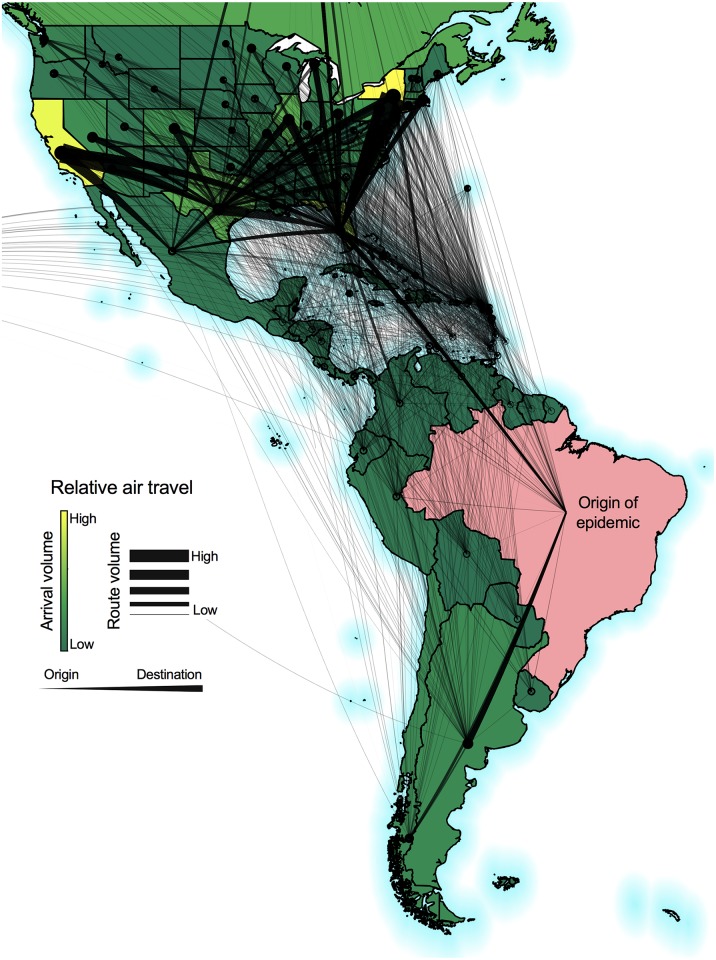
International air passenger travel used to construct the Zika virus epidemic model network. Passenger air travel from regions that reported Zika virus transmission was used to construct a network for potential international virus spread from the epidemic origin, Brazil (pink). The monthly travel volumes were normalized to fit between 0 and 1 and summed across the 18 month study period. The color gradient represents the relative arrival volumes at each destination. The weighted lines represent the travel volumes along each route with the thicker end pointing towards the destination. The travel routes connect region centroids, not specific air ports. We only used departing flights from Brazil (pink). The maps were generated using open source shape files from Natural Earth (http://www.naturalearthdata.com/).

The network upon which the model is implemented is built using passenger air travel data from the International Air Transport Association (IATA), which included origin, destination and stopover airports for all routes, as well as the calibrated passenger travel volumes for each route in the world at a monthly timescale, and represented in Fig 2. The raw airport level data is aggregated to a regional network for the purposes of this study, which includes individual U.S. states, countries, and overseas territories in the Americas. The U.S. states were modeled individually due to the large heterogeneity amongst them, mainly regarding travel patterns, environmental conditions and reported Zika cases. The route-specific passenger travel volumes supplied by IATA were calibrated based on data from 240 airlines comprising 84% of global air traffic, and includes over 3400 airports. The passenger volumes were available at a monthly temporal resolution, which match the temporal resolution of the model. The transportation data used in this paper were limited to air passenger travel volumes and did not include cargo flights. For this study, we only used routes originating in regions with reported Zika virus transmission. We also did not include arrivals into Brazil because we were investigating the risk factors associated with Zika virus spread starting from this region. The analysis was done using flight paths and travel volumes for all routes in 2015. These were the most recent data available for this annual period at the time of analysis, and previously used in [39].

#### Mosquito suitability models

The risk for local Zika virus transmission depends fundamentally on the presence of vector mosquito species, which is a function of environmental suitability. A quantitative relative measure of suitability defines the relative ecological risk for mosquito-borne transmission [44–46]. If the ecological risk is low, local transmission of Zika virus is highly unlikely. If the risk is high, then other factors, such as the the size of the founder vector population and the availability of hosts, become critical for local outbreaks.

**Fig 3:**
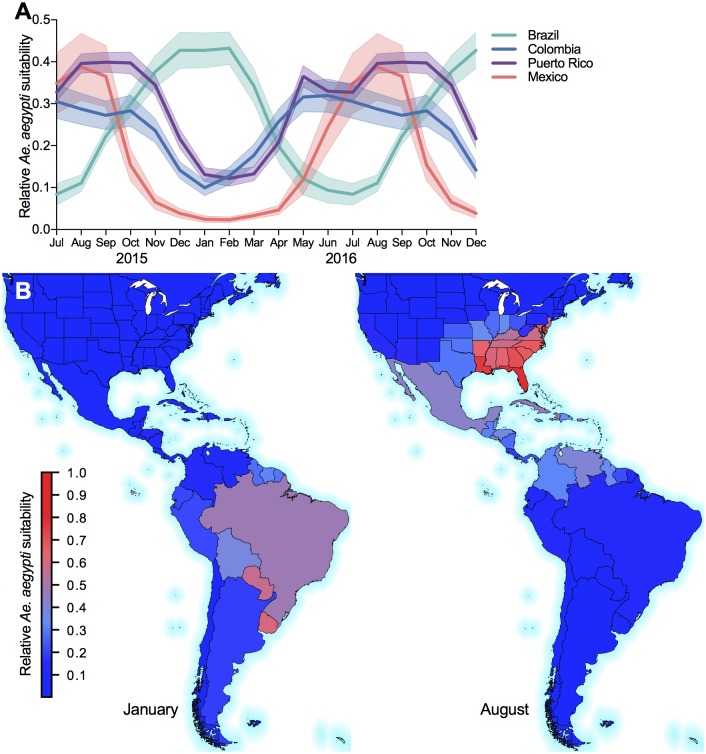
Seasonal and geographical dynamics of *Aedes aegypti* suitability. (A) Monthly mean and standard deviation of relative *Ae. aegypti* suitability for four prominent countries with known local Zika virus transmission. (B) Geographical distribution of mean relative *Ae. aegypti* suitability during periods of Zika virus transmission in the Southern and Northern Hemispheres (January and August, respectively). The maps were generated using open source shape files from Natural Earth (http://www.naturalearthdata.com/).

This analysis is partially based on habitat suitability for the principal Zika virus vector species, *Ae. aegypti*. The monthly vector suitability data sets are based on the original high resolution maps that we previously developed [47] and used elsewhere [3]. We extended our estimates to account for seasonal variation in the geographical distribution of *Ae. aegypti* by using monthly time-varying covariate data including temperature persistence, relative humidity, and precipitation, as well as static covariates such as urban versus rural land use. The newly computed monthly suitability values were rescaled so that the sum of all monthly maps equaled the annual mean. Maps were produced at a 5-km × 5-km resolution for each calendar month and then aggregated to the level of the U.S. states and countries and territories used in this study. These expectations define the relative ecological risk for Zika virus transmission in each cell. Both the mean and standard deviation of the monthly suitability are considered in this study. Fig 3 illustrates an example of the annual fluctuation in suitability by month for four of the countries most impacted by Zika. The effect of seasonality is evident for all countries by the peaks in summer and troughs in winter; the line for Brazil is shifted, due to the difference in seasonality in the southern hemisphere. The figure also illustrates the geographical distribution of mean relative *Ae. aegypti* suitability during periods of Zika virus transmission in the Southern and Northern Hemispheres (Fig 1B, January and August, respectively).

The level of spatial aggregation in the model requires the use of the an average regional suitability value. For some large regions with high variability in environmental conditions and landscape, a mean value is not necessarily appropriate. For this reason we explored the inclusion of the standard deviation of suitability for each region as an additional attribute to capture the existence of high suitability “hot spots”, and thus, still pose a potential risk for transmission even when overall suitability is relative low. However, the variable was excluded in the final analysis because of its poor performance, likely due to the low variability of suitability in many of the smaller Caribbean Islands, where Zika virus was quite prevalent.

#### Socioeconomic and human population data

**Fig 4:**
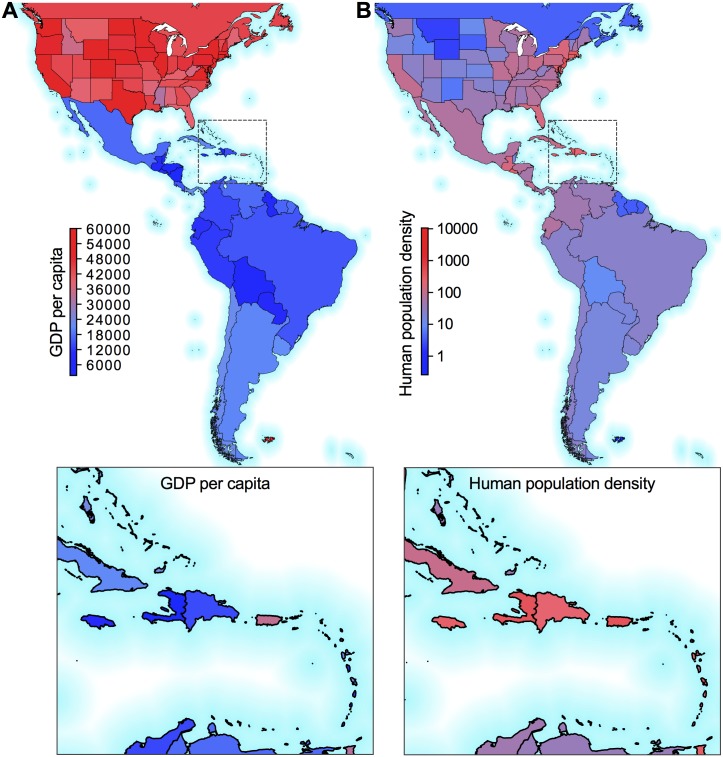
Regional variability in socioeconomic and human population data in the Americas. Regional values for (A) gross domestic product (GDP) purchasing power parity rates per capita and (B) human population densities (people per sq. km of land area) used in the models. The boxes show zoomed in views of the Caribbean Islands. The maps were generated using open source shape files from Natural Earth (http://www.naturalearthdata.com/).

A country’s ability to implement a successful surveillance and vector control programs are critical components to preventing and/or managing an outbreak (if introduced). There is no globally available data to quantify vector control at the country level, therefore alternative economic status and health related indicators were chosen to act as a proxy for available control resources. Economic development is measured by the GDP converted to international dollars using purchasing power parity rates and divided by total population (GDP PPP per capita). GDP data was collected from Worldbank [42] and the U.S. Bureau of Economic Analysis [48], and illustrated in Fig 4. The two variables selected to represent the availability of health infrastructure are: 1) the number of hospital beds and 2) the number of physicians per 10,000 people. Health indicators for the U.S. were collected from the Centers of Disease Control and Prevention (CDC) [49], and data for most regions in the Americas was collected from the WHO World Health Statistics report [50] and from the PAHO [51].

Human population densities (people per sq. km of land area) for each region were collected from Worldbank [52] and the U.S. Bureau of Economic Analysis [48] (Fig 4B).

#### Maps presenting geographical data

The maps presented in our figures were generated using open source shape files from Natural Earth (http://www.naturalearthdata.com/), and rendered using Matplotlib [53]. The software and basemaps are open source and freely available to anyone.

## Method

For the purpose of estimating the risk of Zika virus spread and local transmission in the Americas, we expand upon a previously developed method based on the GIIM [40, 41]. The GIIM model is referred to as an inverse infection model because, instead of attempting to simulate an infection process directly, it uses observations from an actual outbreak on a network and seeks to estimate the parameters of this process, *e.g.,* transmission probability functions on the links of the network. This objective is complementary to a related problem which seeks to infer the set of links most likely responsible for explaining an observed transmission process [54–56]. To accomplish the prescribed task, GIIM relies on information such as the status of confirmed local transmission for each node (or a subest of nodes) in the network, as well as the structure and properties of the network itself. The transmission status is used as a reference point, and GIIM sets the edge weights such that the underlying stochastic simulation model replicates the actual outbreak scale as closely as possible, *i.e.,* GIIM seeks to minimize the error between the observed and reference point. The GIIM model was originally designed for static environments, and is extended to handle dynamic inputs of the application proposed in this work. Both the original model and the extensions are discussed in detail in this section.

### Model inputs

The inputs for the GIIM consist of the underlying network structure, attributes of both the nodes and links of the network, and observations on the actual transmission process. In this work the network structure is defined by the passenger air travel movements between regions in the Americas. We denote graph *G* as *G*(*V*, *E*), where *V*_*G*_ is a set containing all the vertices of the graph, while *E*_*G*_ contains all the edges of the graph. Edges are denoted as *e*_*u*,*v*_, where *u* is the source and *v* is the destination of a travel route represented by the edge. In this paper, *V*_*G*_ contains all countries in the Americas, the French, Dutch, British and American overseas territories, and the individual states of the US, which also defines the spatial resolution of the model. The temporal resolution of the model implemented in this work is at a monthly level, which corresponds to the available travel and suitability data. The edges of the graph exists between a pair of regions if there were passenger air travel movements between them during any month in 2015. The travel patterns between countries is asymmetric, therefore the resulting graph is directed. We denote this graph as *G*_*A*_.

We assign a number of attributes to the vertices and edges of the graph to capture the potential risk factors that may influence the regional spread of Zika virus over time. These attributes correspond to the variables described in the data section. The attributes take the form of normalized real values between zero and one, and include the following:

1. $V_{uv}^{t}$ denotes the normalized monthly passenger air travel volume between regions *u* and *v*, where *u* is the origin and *v* is the destination, and *t* is the month.
2. $I_{u}^{t}$ denotes the Zika incidence rates computed from (suspected and confirmed) cases reported at each month *t* in the origin region *u*, and the population at the origin.
3. $S_{v}^{t}$ denotes the average monthly suitability value for *Ae. aegypti* at the destination *v*, for month *t*.
4. $SV_{v}^{t}$ denotes the standard deviation (STD) of monthly suitability value for *Ae. aegypti* at the destination *v*, for month *t*.
5. *E*_*v*_ denotes the normalized GDP on purchasing power parity (GDP PPP) at the destination *v*.
6. *H*_*v*_ denotes the normalized health indicator at the destination region *v*.
7. *P*_*v*_ denotes the population density of the destination region *v*.

A subset of the attributes are dynamic, *i.e.* their value changes over time, and therefore are denoted with an additional time index, *t*. The dynamic attributes include the passenger volumes, the incidence rates in each region, and the vector suitability. The monthly airport travel volumes, $V_{uv}^{t}$, are aggregated to the state and country level. The mean and standard deviation of the vector suitability for each region for each month is computed as described in the data section. The remaining attributes are static, and are assumed not to vary over the course of the year.

The GIIM defines a transmission model on the input graph. Graph-based transmission models require a real value $w_{e}^{t}\in \left[0,1\right],e\in E_{G_{A}}$ to be present on all edges of the graph, these are called edge transmission probabilities. In this application of the GIIM, the attributes are incorporated into a functional form to represent each time-dependent edge transmission probability. The function takes the form as shown below:

$$\begin{array}{c} w_{e}^{t}=C+\alpha V_{uv}^{t}+\beta I_{u}^{t}+\gamma S_{v}^{t}+\delta SV_{v}^{t}+\omega E_{v}+\rho H_{v}+\lambda P_{v} \end{array}$$ (1)

The variables in the function correspond to the set of attributes previously listed, and the coefficients of each attribute are the parameters to be estimated by the model. Values $w_{e}^{t}$ are bounded between 0 and 1. We will denote the surjective assignment of edge transmission probabilities to the edges as $W_{G_{A}}:E_{G_{A}}\mapsto \left[0,1\right]$.

In addition to the network attributes, GIIM requires a reference observation of the real-life transmission process it seeks to estimate. In the current application, the observation used is the date of the first reported local Zika cases in each region for the time period considered. The reference point is therefore given as a set of 18 binary vectors; each binary vector corresponds to a month of the observed Zika virus outbreak starting from July 2015 to December 2016, and assigns a value of 0 or 1 to each node of the graph indicating its transmission state. A value of 1 indicates that the presence of local transmission of Zika virus in the region was reported within or before the corresponding month, and a value 0 indicates that Zika cases have yet to be reported from the region.

### Stochastic simulation model

The GIIM relies on an underlying stochastic simulation to model the spreading process. The compartmental model that is used in this paper is the SI model, which has two states: susceptible (S) and infected (I). The graph-based SI infection model is an iterative discrete-time model that assigns states to the nodes of the graph: each node can only be in a single state at any time step, and all nodes must have a state at all times. While GIIM can accommodate a more complex compartmental model, *e.g.,* SEIR, the SI model is selected to fit the Zika virus application based on the assumption that once Zika virus becomes locally established, that region remains a potential risk of furthering the spread of the virus for the time frame being considered. The reason for allowing the option of non-zero outgoing risk after the reported case count reduced to zero is that there could still be infected individuals in the region, especially given the high rate of asymptomatic cases and reporting error. However, it is important to note that the use of the SI model does not enforce a region to have a positive transmission probability over the entire period modeled, it simply allows a non-zero transmission risk value to be estimated by the model. The actual time-dependent transmission probability is defined as a function of the incidence rate at the origin country among other factors; a probability of zero is a feasible solution of the model, and is actually assigned to many of the links over the course of the outbreak. Given the estimated transmission probabilities, in each step of the simulation, “infected” nodes try to “infect” all their susceptible neighbors according to the transmission probability *w*_*e*_ connecting them. If the attempt is successful, the neighbor will be infected in the following iterations. If the attempt is unsuccessful, the neighbor remains in a susceptible state, and the infected node can continue to make attempts in the following iterations indefinitely.

More formally, the transmission process starts from an initially infected set of nodes $A_{0}\subset V_{G_{A}}$ at iteration *t*_0_. The rest of the nodes $V_{G_{A}}\backslash A_{0}$ are susceptible at the beginning of the process. Let $A_{i}\subseteq V_{G_{A}}$ be the set of infected nodes at iteration *i*. At each iteration, *t*, each node *u* ∈ *A*_*i*_ tries to infect each susceptible neighbor $v\in V_{G_{A}}\backslash A_{i}$ according to the probability $w_{e}^{i},e=\left(u,v\right)\in E_{G}$. If the attempt is successful, *v* becomes infected starting from the following iteration: *v* ∈ *A*_*i*+1_. If more than one node is trying to infect *v* in the same iteration, the attempts are made independently of each other in an arbitrary order within the same iteration. By definition, the transmission process stops at iteration *t* if $A_{t}=V_{G_{A}}$. Since we only simulate a finite portion of the Zika virus epidemic, we stop all transmission models after 18 iterations, corresponding to the observed months of the epidemic at the time of analysis.

Within each model run, the transmission process is repeated 10000 times to produce a real value indicating the likelihood of each node being in an infectious states at each iteration, *t*. The value is calculated by counting the number of repetitions when nodes were in an infectious state, and dividing by the total number of repetitions *i. e.*10000.

### Generalized inverse infection model solution methodology

The GIIM [41] implemented in this work formulates the estimation of edge transmission probabilities as a general optimization task. The model relies on knowledge of the underlying graph and (at least a subset of) observations from a transmission process taking place on the network. The real observations take the form ${\vec{o}}_{t}\in O$, where $\vec{o}$ is a binary vector and *t* is a time stamp. Each vector represents a point in time and ${\vec{o}}_{t}$ assigns a binary value to all $v\in V_{G_{A}}$ indicating the observed transmission (or lack there of) of Zika virus in the region at time *t*. Set *O* contains all observations, while set *T* contains all sample times, *i.e.* |*O*| = |*T*| = 18.

The inputs of GIIM are: an unweighted graph *G*, a transmission model I, the set of sample times *T*, and the set of observations *O*, where O=Inf(G,W,I,T). In this work I is the SI stochastic simulation transmission model defined in the previous subsection, *W*_*G*_: *E*_*G*_ ↦ [0, 1] is the unknown weight assignment, and *Inf* is a procedure that generates observations at sample times *T* based on transmission process I taking place on graph *G* with assigned edge weights *w*_*e*_ ∈ *W*, *e* ∈ *E*(*G*). The set of observations, *O*′, is a time-dependent vector of real values equal to the probability each node is infected at each timestep, computed from the set of runs. The task of GIIM is to find an estimation *W*′ of *W* so that the difference between *O* and $O^{\prime }=Inf\left(G,W^{\prime },I,T\right)$ is minimal. Due to the need to compare a set of binary vectors with a set of real vectors, we compare observations *O* and *O*′ using ROC evaluation by pairwise comparing vectors ${\vec{o}}_{i}\in O$ and ${\vec{o^{\prime }}}_{i}\in O^{\prime }$ for *i* = 1…18, computing the AUC value for each pair and averaging over all pairs.

The formal definition of the GIIM is as follows:

**General Inverse Infection Model:** *Given an unweighted graph G, and transmission model* I, *the set of sample times T and observations* O=Inf(G,W,I,T), *we seek the edge transmission probability assignment W*′ *such that the difference between O and* $O^{\prime }=Inf\left(G,W^{\prime },I,T\right)$ *is minimal*.

The GIIM defines the estimation of *W* as an optimization problem, which is solved using an iterative refinement algorithm. The procedure begins with an initial weight configuration $W_{0}^{\prime }$, runs transmission model I, makes observations *O*′ and computes the error between *O* and *O*′. Based on the error, *W*′ is refined and the process is repeated, until the error becomes less than an accuracy constant *a* selected by the user. The search strategy used in this paper is the Particle Swarm Optimization (PSO) method [57]. According to the findings in [41], the method is stable and is able to produce outputs close to the reference, and therefore the solution method chosen for this work as well. It can also be implemented in a parallel environment speeding up computations considerably. Algorithm 1 summarizes the iterative GIIM algorithm.

**Algorithm 1 Generalized Inverse Infection Model**

1: Inputs: G, I, T, O, a

2: Choose initial edge infection probability assignment $W^{\prime }$

3: **repeat**

4:  Compute $O^{\prime }=Inf\left(G,W^{\prime },I,T\right)$

5:  Compute $d(O,\;O^{\prime })$

6:  **if** $d(O,\;O^{\prime })\;\leq \;a$ **then**

7:   **return** $W^{\prime }$

8:  **else**

9:   Choose new $W^{\prime }$ according to the PSO search strategy.

10:  **end if**

### Dynamic extension of general inverse infection model

A major modification was necessary in order to adapt the GIIM method to be applied in the context proposed here. The original GIIM method estimates the edge transmission values of the graph directly as real values that are static, *i.e.,* they do not change over time [41]. In this work the edge weights are given as functions of known attributes on the nodes and edges of the graph, and the task becomes the estimation of these functions, or more specifically, the coefficients of these functions. Several attributes in this application are dynamic, *i.e.,* they change with time. Thus, it is necessary to further extend the function estimation method to account for dynamic attributes, and to adapt the simulation model to handle dynamic edge transmission values.

In this work the edge transmission values are defined using a linear function of known attributes, as defined in eq (1). More generally, if $a_{i}^{t}\left(e\right),e\in E_{G_{A}}$ is the set of attributes, where *i* represents the *i*-th attribute and *t* represents the time period, then the time-dependent edge transmission probabilities $w_{e}^{t}$ are given as $w_{e}^{t}=g\left(f_{1}\left(a_{1}^{t}\left(e\right),\vec{c_{1}}\right),f_{2}\left(a_{2}^{t}\left(e\right),\vec{c_{2}}\right),\ldots ,f_{\ell }\left(a_{\ell }^{t}\left(e\right),\vec{c_{\ell }}\right),\vec{c_{g}}\right)$ for all $e\in E_{G_{A}}$, where *ℓ* is the number of available attributes, *f*_1_, …, *f*_*ℓ*_ and *g* are functions and $\vec{c_{1}},\ldots ,\vec{c_{\ell },\vec{c_{g}}}$ are coefficients of functions *f*_1_, …, *f*_*ℓ*_, *g*. Following the notation proposed in [41], the functions used to compute the edge transmission probabilities are given as a set of attribute functions *f*_1_, …, *f*_*ℓ*_ assigned to each invididual attribute, an aggregator function *g* with the role of creating a single value from the result of the attribute functions and a normalization function ensuring that the result falls between 0 and 1. This formulation makes implementation of the method easy while retaining the flexibility of the model. Let *C* denote the set of all coefficient vectors. The values of the attributes can change over time, however the functional form and estimated coefficients are assumed to remain constant over the time period examined.

The optimization process of GIIM changes from the estimation of the direct weight assignment *W* to the estimation of *C*. This provides a means to identify the key factors contributing to the spread of Zika virus throughout the regions in the Americas. A second advantage is technical; |*C*|<< |*W*|, therefore the optimization process is much easier because we are only looking for a limited number of function coefficients as opposed to the edge transmission probabilities for all edges of the graph. For the implementation of the model in this paper, all parameters are bounded between -0.5 and 0.5, in order to reduce the solution space for the PSO method. Finally, the resulting edge transmission values are trimmed above 1 and below zero by taking $w_{e}^{t}=MAX\left(0,MIN\left(1,\sum_{i=1}^{\ell }f\left(a_{i}^{t}\left(e\right),c_{i}\right)+c_{g}\right)\right))$.

## Results and discussion

By the time Zika virus was first detected in Brazil in May, 2015, the virus had already rapidly spread to most regions of the Americas [3, 4]. The goals of our analyses were 1) to identify the relative contribution of each risk factor in the spread and local outbreaks of Zika virus and 2) to compute the risk of spread (or re-introductions) between each pair of regions during 2016. We aggregated the route level risks to provide a relative ranking of total importation and exportation risk posed by and to each region per month during the outbreak. Our final network, representing feasible air travel routes in the Americas, is a directed, weighted graph structure with 103 nodes and 2946 edges.

### Estimated contribution of risk factors

**Fig 5:**
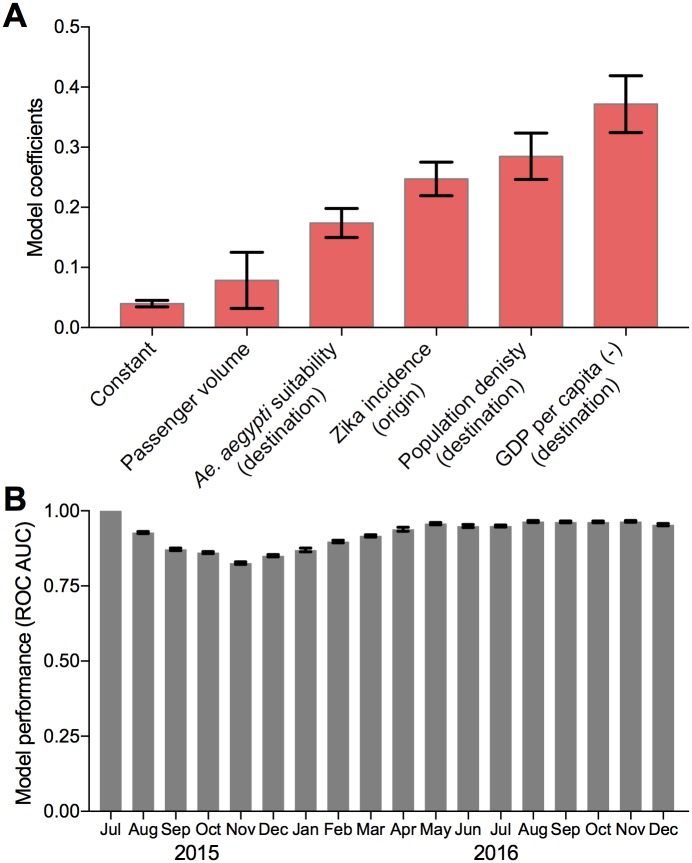
Estimated risk factors for Zika virus transmission and spread. (A) The mean and standard deviation of the model coefficients estimated across 20 runs. The estimated coefficients represent the relative contributions of each risk factor. (B) The model performance mean and standard deviation values for each month of the observations period.

The first task of our study is to identify the set of attributes (and corresponding contribution of each) to be included in the model. We consider the entire set of attributes previously presented in the data section. A linear weighted sum function as defined in eq (1) is used to compute transmission probabilities, and the dynamic GIIM method is implemented to estimate the coefficients of the function. Different variable configurations were considered, and the model that produced the best fit was selected. The model fit is based on the quantifiable performance metric, ROC AUC averaged over the entire time period. To evaluate and ensure stability of the proposed estimation method, we ran the algorithm 20 times with the same set of inputs and computed the mean and variance of the estimated model coefficients over all runs. The results of the final model are presented in Fig 5.

$$\begin{array}{c} w_{e}^{t}=0.040+0.079V_{uv}^{t}+0.247I_{u}^{t}+0.174S_{v}^{t}-0.372E_{v}+0.285P_{v} \end{array}$$ (2)

The results of the model are highly robust. The estimated coefficients vary minimally across runs, and, more significantly, the ranking of all risk factors remained consistent across all runs (Fig 5A). The expected value of the estimated coefficients represent the relative influence of each attribute in the risk of Zika virus spread between a pair of regions. When interpreting these coefficients it is important to first note that this model is designed to estimate the risk of Zika virus spread between two regions *resulting in local, vector-borne transmission*. This is different from the risk of Zika infected passenger arrivals. For example, there are multiple locations where travel cases of Zika were continually reported, yet no local cases resulted [27]. The lack of local transmission in these examples could be due to many explanations, including insufficient populations of competent vectors and/or intense surveillance and vector control programs implemented at the destination. Thus, a high number of travel-reported cases does not necessarily translate to a high local transmission risk, and for this reason, the risk of Zika infected passenger arrivals and local transmission risk should be modeled separately. Due to the potential harm posed by local outbreaks of Zika, local transmission risk is the primary focus our analyses.

The important distinction between modeling travel-reported cases and local transmission risk is perhaps most evident from the low coefficient estimated for travel volume. In fact, we found infected travelers to be significantly less influential than all the other risk factors. This can be explained by the fact that there is a high level of connectivity between most pairs of regions, and more importantly, the highest volume of air travel exists between and within the U.S. states. Yet, with the exception of Florida [24] and Texas [58], Zika was not broadly established in the U.S. Thus, we do not identify travel volume to be a driving forces in the spread and transmission of Zika virus in the Americas; travel is a necessary, but not sufficient condition. It is worth noting that a model seeking to estimate the risk of infected passenger arrivals alone would likely find this variable to play much more substantial roles.

In contrast, we found that Zika virus spread and local transmission is largely driven by regional attributes at the origin (incidence rate) and destination: *Ae. aegypti* suitability, human population density, and the GDP, with GDP being the most significant (Fig 5A). As expected, a higher incidence rate at the travel origin, which can act as a proxy for the likelihood of an infected individual arriving at the destination, significantly increases the risk posed to the travel destination. Similarly, the results indicate a higher vector suitability at the destination corresponds to an increased risk of transmission. High human population density at the destination is also revealed to increase the risk of transmission, consistent with the required presence of both vectors and hosts for mosquito-borne virus transmission. The health indicator variables were excluded from the final model, as they were not found to have a significant impact.

Based on our model results, the most dominant and only negative risk factor is a region’s GDP, *i.e.,* a higher GDP at the destination corresponds to a lower risk of transmission. Based on the magnitude of the coefficient, the destination’s GDP contributes more than any other risk factor considered. The highly negative coefficient of GDP can be explained by the substantial delay (or complete lack) of local transmission in the wealthier U.S. states and certain territories and islands in the Caribbean. GDP is obviously not directly involved in Zika virus transmission, but it may indirectly influence the interactions between components of the cycle: hosts and vectors. Poorer nations likely have lower housing qualities and inhabitants may be exposed to more mosquito bites, *e.g.,* a lack of screens on windows and doors allowing mosquitoes to enter. They may also have more debris around their homes acting as breeding containers for *Ae. aegypti*. Lastly, GDP may be a proxy for the available surveillance and vector control resources at the destination, an increase of which would aid in reducing local transmission.

Our final model captures the relative contribution of both static and dynamic risk factors to explain the spread and local transmission of Zika virus in the Americas. The AUC ROC for this model averaged over the 18 months is 0.923, which indicates an excellent fit (Fig 5B). For the first month we see perfect classification, since the epidemic was only reported in Brazil, which proves trivial for the estimation method. The second half of 2015 corresponds to a major increase in the number of regions reporting local transmission (as illustrated in Fig 1). Specifically, between November and December the number of regions reporting transmission nearly doubled from 9 to 16, and then increased to 32 in January. This proved to be the most challenging part of the estimation process, and the reason for the performance drop below 0.9 during the period from October to January. The minimum AUC value of 0.83 occurred in November 2015, which is still considered to be good performance for any classifier. The estimation task gets easier again for the last 9 month characterized by a low but constant rate of spreading between the regions, and performance goes around 0.95 again for these months. We can conclude, that even though sudden bursts in the reported local outbreaks decrease prediction accuracy slightly, the method is able to provide accurate estimation. The estimation process took between 3 to 4 hours with the number of iterations between 250 and 350. A parallel version of the algorithm was implemented in C++, and the results were computed on a PC with an 4-core i7-7700k 4,2 GHz processor.

### Route level risk

**Fig 6:**
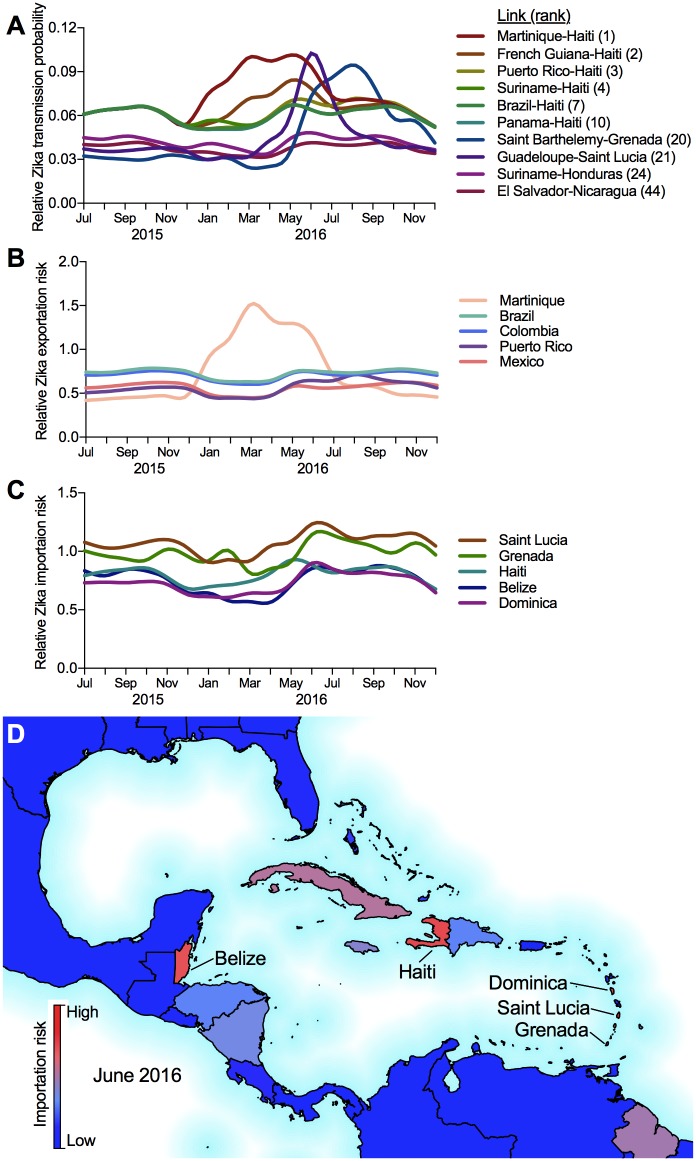
Temporal and regional risks for Zika virus introductions and transmission. (A) The temporal probabilities for Zika virus introductions via infected travelers and establishment of local transmssion are shown for 10 links, listed as origin-destination (rank). Relative risk profiles for temporal and regional Zika virus (B) exportations and (C) introductions were estimated using the network-level transmission probabilities (top 5 ranked regions shown for each). (D) Geographic variation in relative Zika virus importation risk for June, 2016. All Zika virus transmission data can be found in S1 Data. The maps were generated using open source shape files from Natural Earth (http://www.naturalearthdata.com/).

Using the final estimated model, we computed the probability of Zika virus spread between any pair of regions in the Americas resulting in subsequent local transmission (Fig 6). The link probabilities reveal the highest risk travel routes connecting regions at discrete points in time over the outbreak, as well as how the relative ranking changes over time (Fig 6A). Out of the 2946 feasible edges in the network, only 711 edges have nonzero transmission probabilities, and only 58 of the edges has a value greater than 5% at any point of the observation period. The time-dependent data for the top 100 transmission links, exportation risk, and importation risk are provided in S1 Data.

In general, the high risk travel routes are dominated by ones outbound from the Caribbean Islands with the highest incidence rates (earlier in the outbreak), and pointing to the less developed countries and territories of the Caribbean and Central America. These results are corroborated by the early estimated introduction times into countries like Haiti and Honduras relative to Puerto Rico, Mexico, and the U.S. [3, 4, 24], see also www.nextstrain.org/zika. The risk on routes departing a given country, *e.g.,* Brazil, behave similarly, but vary in magnitude. They also display different behavior than the outgoing risk posed by other high risk countries, *e.g.,* Martinique. The estimated transmission probabilities fluctuate over time due to changes in the dynamic attributes at both the route origins (outbreak scale) and destination (vector suitability), as well as variations in the monthly travel volumes between regions. The effect of these risk factors, for example, the lower Zika incidence rates in Puerto Rico relative to Martinique (Fig 1B), is illustrated by the corresponding temporal differences in transmission risk into Haiti (Fig 6A).

The route level risk can be aggregated to provide import and export risk profiles at a regional level. This type of spatial and time-dependent information can help guide policy decisions, such as where to allocate available resources at different stages during an outbreak. The regional level risk is achieved by computing the node strength statistic for all nodes of the network. Node strength is defined as the sum of all weights incident to a node. In the case of a directed network, out-strength, *i.e.,* the sum of all outgoing link weights, and in-strength, *i.e.,* the sum of all incoming link weights, are calculated to provide the relative export and import risks. Node strength values can be used to rank the regions according to outgoing (export, Fig 6B) and incoming risk (import, Fig 6C and 6D), and more critically, observe how the ranking and magnitudes of the risk change over the course of the outbreak.

### Regional exportation risk

To determine the regions most likely to contribute to the spread of Zika virus during the epidemic, we estimated the the dynamic exportation risk using the route-level network (Fig 6B). Martinique, Brazil, Colombia, Puerto Rico and Mexico are identified by the model to pose the highest risk of spreading Zika to new regions. Intuitively, the export risk is dominated by the set of counties infected earlier in the outbreak and those with high incidence rates. Martinique stands out as having the highest exportation risk, which peaks during March, corresponding to the month with the highest incidence rate (Fig 1B). The highest exportation risk through 2015 is posed by Brazil and Colombia, which were the two countries reporting early and large outbreaks (Fig 1A). Brazil was identified as the likely source of spread to the first few Caribbean Islands, which is consistent with the phylogenetic data [3, 4]. Our model estimates that Brazil and Colombia became less prominent in their roles of seeding new Zika virus outbreaks from December, 2015, to May, 2016. This time period corresponds to the significant rise in the number of new regions reporting local Zika virus transmission (Fig 1C) and the rise of Zika incidence rates in many Caribbean Islands (Fig 1B). The increased exportation risk posed by Martinique during this time captures this behavior (Fig 6B).

### Regional importation risk

We similarly aggregated the route-level network at each destination to determine temporal and regional importation risk (Fig 6C and 6D). The regions at highest risk of local transmission are dominated by the less developed countries and territories in Central America such as Belize, and the islands in the Caribbean, such as Haiti, Saint Lucia, Grenada, and Dominica. The high ranking of these regions is due to their low GDP (Fig 4A), high human population density (Fig 4B), and high vector suitability (Fig 3).

For the U.S., the states with the highest importation risks were Florida, Georgia, and South Carolina, mostly due to their high *Ae. aegypti* suitability (Fig 3) and incoming travel volume from the affected regions [24]. Compared to much of the Americas, however, these importation risks were low, predominantly due to their high GDP (Fig 4A). In fact, Florida was the only one of those three states (along with Texas) that reported local Zika virus transmission. These findings correspond with and reinforce previous route-level risk rankings, that is less developed regions are more likely to see local Zika virus transmission, given they meet the basic requirements for *Ae. aegypti*-borne virus transmission.

### Sensitivity to case reporting errors

**Fig 7:**
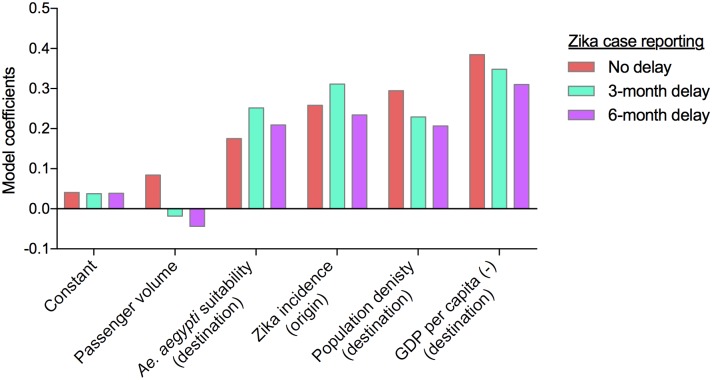
Sensitivity analyses accounting for delays in reporting Zika virus transmission. The model was re-run by shifting the reported Zika virus cases by three and six months for each region to account for delays in outbreak detection.

Much of the dynamic aspects of our network model are based upon reported Zika virus cases, which are likely biased and vastly underestimated. Moreover, there is often a substantial delay (3-12 months) between actual introduction of the virus and the first reported case [2–4, 24]. The reporting inaccuracies can be attributed to the high percentage of asymptomatic Zika cases and insufficient surveillance methods, among other factors. While we cannot feasibly correct for all reporting inaccuracies, we conducted sensitivity analyses to account for delays in case reporting (Fig 7). Both three- and six-months reporting delays were considered, and the model was re-run with corresponding shifts in the data to represent each assumption. While the coefficient rankings slightly changed, likely representing better fits between local vector suitability and Zika incidence rates, the general conclusions were unchanged—low GDP was still the best predictor of local Zika virus transmission. Thus, our results appear to be robust to some reporting inaccuracies.

### Limitations

Our work takes a major step towards improving our understanding of the risks associated with Zika virus spread and local transmission; however, there are certain limitations of this analysis that must be noted here and addressed in future research:

1. For consistency, we aggregated all attributes to the same spatial scale, and due to limitations on available case data, the model was implemented at the state and country level. For some of the smaller countries and Caribbean islands with only one major city or international airport, this was not a major problem. For large countries with substantial heterogeneity across the region, however, certain aggregated attributes are not necessarily representative. The mean vector suitability is perhaps the most compromised attribute. The use of the an average suitability across a large region with high variability in environmental conditions and landscape is not accurate, and may result in underestimations or overestimations of potential risks for certain regions included in the model.
2. Reported Zika case data is vastly underreported and subject to substantial variability across regions. This issue affects two attributes used in this model, the incidence rates and the dates of the first reported cases. The level of inaccuracy is impossible to quantify. However, we partially addressed this issue through sensitivity analysis conducted around assumed reporting delays (Fig 7). Our analysis demonstrates that reporting delays would not likely alter our general conclusions regarding the risk factors associated with Zika virus spread.
3. Due to the lack of case data for Brazil between May and December 2015, estimated case counts were used (as described in the data section). These estimated values may result in an overestimate or underestimate of the actual number of cases during this time period, and potentially impact the estimated outgoing risk posed by Brazil.
4. At the start of this study, 2015 monthly air travel data was the most recent data available and was therefore used in place of 2016 data in the analysis. The main discrepancy due to this substitution is in August, when the 2016 Olympics where held in Rio. The travel patterns into and out of Brazil were likely substantially different in 2016, and would not be represented by the 2015 data set. Even with 2016 IATA data, however, the actual travel patterns surrounding the Rio Olympics would not be captured because a large portion of the trips were made using chartered flights, which are not recorded in the same data set. Furthermore, in August 2016, the number of local cases reported in Brazil dropped substantially (Fig 1A), as did the number of travel reported cases from Brazil during and immediately succeeding the Olympics.
5. This study accounts for a single mode of inter-regional travel, passenger air trips, and excludes land and sea travel. Due to these limitations, the results from this analysis likely underestimate the risk posed to destinations in close spatial proximity to affected regions, as well as the risk posed to the gulf U.S. states and Caribbean Islands with substantial cruise ship travel [24]. To accurately capture multi-modal human mobility patterns requires data from multiple transport sources or data which is not linked to any specific transport mode. Mobile phone data, as one such option, offers significant potential in modeling and predicting epidemic spread [39].
6. Zika virus could be efficiently spread by mosquito species other than *Ae. aegypti*, which is not accounted for in this study. The potential role of *Ae. albopictus* in transmitting Zika virus poses additional risk because it has a much wider presence in temperate regions, including southern regions of the Americas and Australia, as well as the northern United States and parts of Europe and Canada [38, 47]. However, we recently presented data to suggest that *Ae. aegypti* presence alone best explains the geographical distributions of Zika virus outbreaks in the Americas [39], thus the reason for it being the focus in this work.
7. Finally, this analysis ignores the role of potential non-vector-borne mechanisms of Zika virus transmission. This includes sexual [59–61] and vertical transmission [8]. While vector habitat suitability will likely continue to play a predominant role in the spread of Zika virus, further research is necessary to understand how alternative transmission mechanisms may impact the local risk profiles.

### Conclusions

Our work enhances our understanding of and ability to investigate the risk factors which contributed to the spread and local transmission of Zika virus during the 2015-2016 epidemic in the Americas. For each region, our model is informed by data on regional socioeconomic factors, vector habitat suitability, passenger air travel data, and epidemiological data. We constructed and implemented a dynamic extension of the GIIM to estimate the contribution of each risk factor to the likelihood of Zika virus transmission. Our model relies on a multi-agent based optimization method to estimate the parameters and a data driven stochastic-dynamic epidemic model for evaluation. The GIIM was shown to perform well based on quantitative metrics.

Our results from the final model indicate the spread and local transmission of Zika virus was quite multifaceted. As expected, regional attributes influencing vectors (*Ae. aegypti* suitability), hosts (human population density), and viruses (Zika incidence rates at origin of travel) all contributed to the likelihood of establishing local mosquito-borne transmission. Passenger air travel volume, however, was notably less impactful that the other attributes. Therefore, rather than travel restrictions, we predict that mosquito control will be more effective at reducing Zika virus introductions leading to local transmission. This debate recently arose during the 2016 Rio Summer Olympics where some wanted to ban the games to prevent further Zika virus spread [62]. Our results suggest that additional travel for the Olympics was highly unlikely to make a significant impact.

From our model, the coefficient most associated with Zika virus transmission was the regional GDP per capita, where a lower GDP corresponded to higher transmission risk. Although GDP does not directly influence transmission, it likely serves as a proxy for mosquito-host interactions [63] and surveillance activities. For example, people living in poverty often do not have the means to protect themselves from host seeking mosquitoes, such as air conditioning and screened windows common in higher income areas. Findings by Netto et al. [64] of higher Zika virus seroprevalence in areas with lower socioeconomic status further support our association. This, now evidence based conclusion that Zika and other *Ae. aegypti*-borne viruses should be considered diseases of poverty, is also consistent with other expert opinions [37, 65, 66].

The two most significant risk factors identified in our work, namely GDP and population density, are often excluded in geographic risk profiling of *Aedes* vector-borne diseases, and should be considered in future analysis. Our model is not specific for Zika virus and could easily be employed for other mosquito-borne viruses, such as dengue and chikungunya, with sufficient epidemiological and entomological data. Furthermore, the model could be adapted as a tool to inform real-time policy decisions regarding resource allocation for destination-based surveillance and vector control.

## Supporting information

S1 Data The excel file includes the following input data and model results a) Regional population density, GDP and monthly reported case data used as input in the model; b) Monthly Case data for each country; c) The top 100 routes likely to result in local Zika transmission at the destination and their time-dependent relative risk estimates; d) The complete set of regions included in the model and their time-dependent relative importation risk; e) The complete set of regions included in the model and their time-dependent relative exportation risk.
(XLSX)
